# A 6-Membrane Protein Gene score for prognostic prediction of cytogenetically normal acute myeloid leukemia in multiple cohorts

**DOI:** 10.7150/jca.35382

**Published:** 2020-01-01

**Authors:** Sheng-Yan Lin, Ya-Ru Miao, Fei-Fei Hu, Hui Hu, Qiong Zhang, Qiubai Li, Zhichao Chen, An-Yuan Guo

**Affiliations:** 1 Hubei Bioinformatics & Molecular Imaging Key Laboratory, Department of Bioinformatics and Systems Biology, Key Laboratory of Molecular Biophysics of the Ministry of Education, College of Life Science and Technology, Huazhong University of Science and Technology, Wuhan, 430074, China.; 2 Institute of Hematology, Union Hospital, Tongji Medical College, Huazhong University of Science and Technology, Wuhan, 430022, China.

**Keywords:** Cytogenetically normal acute myeloid leukemia, membrane protein genes, MPG6 (6-Membrane Protein Gene) score, risk stratification, outcome prediction.

## Abstract

**Background:** Cytogenetically normal acute myeloid leukemia (CN-AML) is a large proportion of AMLs with diverse prognostic outcomes. Identifying membrane protein genes as prognostic factors to stratify CN-AML patients will be critical to improve their outcomes.

**Purpose:** This study aims to identify prognostic factors to stratify CN-AML patients to choose better treatments and improve their outcomes.

**Methods:** CN-AML data were from TCGA cohort (n = 79) and four GEO datasets. We identified independent prognostic genes by Cox regression and Kaplan-Meier methods, and constructed linear regression model using LASSO algorithm. The prediction error curve was calculated using R package “pec”.

**Results:** Based on independent prognostic membrane genes, we constructed a regression model for CN-AML prognosis prediction: score = (0.0492 * *CD52*) - (0.0018 * *CD96*) + (0.0131 * *EMP1*) + (0.2058 * *TSPAN2*) + (0.0234 * *STAB1*) - (0.3658 * *MBTPS1*), which was named as MPG6 (6-Membrane Protein Gene) score. Tested in multiple CN-AML datasets, consistent results showed that CN-AML patients with high MPG6 score had poor survival, higher WBC count and shorter EFS. Comparing with other reported scoring models, the benchmark result of MPG6 achieved better association with survival in multiple cohorts. Moreover, by combining with other clinical indicators in CN-AML, MPG6 could improve the performance of survival prediction and serve as a robust prognostic factor.

**Conclusions:** We identified the MPG6 score as a stable indicator with great potential for clinical application in risk stratification and outcome prediction in CN-AML.

## Introduction

Acute myeloid leukemia (AML) is a heterogeneous disease characterized by expansion of undifferentiated myeloid precursors, resulting in impaired hematopoiesis 1. Currently, chromosomal aberrations are well established as the diagnostic and prognostic markers in AML 2. Patients with abnormal cytogenetic chromosomes such as PML-RARA, RUNX1-RUNX1T1 or MYH11-CBFB fusions are associated with favorable prognosis, whereas patients with monosomy karyotype or complex alterations are associated with high prognosis risk 3. However, nearly half of AML patients are cytogenetically normal (CN-AML) with an intermediate prognosis and markedly diverse outcomes 4. Therefore, it is very important to identify prognostic factors to stratify CN-AML patients to choose better treatments and improve their outcomes.

Recently, DNA mutations and aberrant RNA expression profiles were proposed as prognostic indicators for the treatment outcome in CN-AML. Notably, recurrent lesions in NPM1, FLT3-ITD and CEBPA mutations were identified to improve risk stratification for CN-AML patients 5. Aberrant expression level of single gene such as *DNMT3B* 6, *BAALC* 7, and *ERG* 8 has also been reported to be associated with CN-AML patient outcome. Meanwhile, prognostic indicators based on multiple genes were also integrated to define CN-AML subgroups. In 2011, an integrative prognostic risk score based on clinical and molecular markers for gene expression and mutation was proposed for outcome prediction of CN-AML patients 9. Recently, high expression of stem cell-associated genes was validated with negative prognostic impact in primary CN-AML 10. However, these prognostic signatures lack consistency in different CN-AML cohorts and are not easy to use because they refer to many genes and mutations. Thus, there was still no stable and easy-used prognostic gene signatures applied into risk classifications in CN-AML. Currently, large scale datasets in TCGA 11 and GEO 12, and their analysis tools such as GSCALite webserver 13 made the analysis feasible.

Membrane proteins are essential for many biological processes such as cell signaling, transporting and cell adhesion and approximately occupy 20-30% of genes in human genome 14, 15. Moreover, membrane proteins represent 60% of the known drug targets for therapeutics 16. Thus, they are ideal biomarkers as an independent predictor for CN-AML patients' prognosis and classification. In this study, we generated a 6-Membrane Protein Gene (MPG6) score that was highly correlated to survival outcomes. The MPG6 score was confirmed to be independent in five CN-AML datasets in overall survival (OS) models. More importantly, we showed that the MPG6 score could improve the predictive performance to predict patients' survival and function as a good prognostic factor.

## Material and methods

### Patient's clinical information

We downloaded the gene expression data and clinical information of 79 CN-AML from the TCGA LAML dataset (https://tcga-data.nci.nih.gov/tcga/). Four other CN-AML microarray datasets (GSE311602 (n = 79) 11, GSE71014 (n = 104) 17, GSE12417 (n = 163) 11 and GSE6891 (n = 187) 18 were downloaded from the GEO database and were normalized as described by Metzeler et al 11. The GSE311602 was a test cohort consisted of 79 adult German patients who were diagnosed with CN-AML in 2004. The GSE71014 cohort consisted of 104 CN-AML patients from the National Taiwan University Hospital. The GSE12417 was a training cohort consisted of 163 adult German patients. The patients in GSE6891 were diagnosed as CN-AML younger than or equal to 60 years. We should note that the clinical information such as age, sex, white blood cell (WBC) count, mutation status and FAB classification in TCGA and GSE6891 datasets were more complete than other three cohorts, which only have the OS information in GEO portal.

### Survival analysis and membrane protein gene identification

High and low gene expression was defined using the median expression level of all CN-AML samples as threshold in that cohort. OS was defined as the time from AML diagnosis until death from any cause or last clinical follow-up. Clinical variables such as age, sex, WBC count, mutation status and FAB classification were assessed in the univariate analysis using the Kaplan-Meier method with the R package “survival” and variables with p-value <0.1 were remained for further analysis. After univariate analysis, we combined those significant prognostic factors in a multivariate analysis. The log-rank test was used to assess statistical significance. For multivariate analysis, multivariate Cox regression model 19 was used to study the association between gene expression levels and OS in the presence of other known clinical covariates such as age, sex, WBC count, mutation status and FAB classification in R package. Hazard ratios (HR) with relative 95% confidence interval (CI) were shown in multivariate analysis. The list of membrane protein genes were from Membranome 2.0 webserver 20.

### Prognosis signature training

The dataset TCGA CN-AML (n = 79) was used for prognosis signature training. The correlation analysis of gene expressions was using R package “psych” (p <0.01, r <0.5) and genes with expression value ≤5 (RPKM) were removed as low expression. Using all genes' expression as features, we performed the linear regression analysis for gene expression against survival based on the Least absolute shrinkage and selection operator (LASSO) algorithm. Next, Sequential Backward Selection (SBS) method was used to choose the optimal feature for the model. Finally, an optimized linear regression model that made up of six membrane protein genes (MPG6) was constructed, which was highly correlated to survival outcomes in the training cohort.

### Prognosis signature testing

We tested the robustness and practice performance of the MPG6 score in other four CN-AML cohorts: GSE311602 (n = 79), GSE71014 (n = 104), GSE12417 (n = 163) and GSE6891 (n = 187). For each cohort, we performed survival analysis with the median threshold of the MPG6 score to separate samples into high and low groups. Specifically, we counted the number of samples whose OS >2 years and OS >3 years in the high and low score groups. Then, we investigated the association between MPG6 score and other clinical indicators such as age, sex, WBC count, FAB classification, the presence of FLT3-ITD mutation and NPM1 mutation in TCGA CN-AML (n = 79) and GSE6891 (n = 187) cohorts.

### Predictive performance test and independent prognostic analysis

The performance of the MPG6 score to predict survival of CN-AML patients was conducted by logistic regression. First, we used the score as the single variant to predict survival. Then, we combined multiple variants including age, WBC count, FLT3-ITD mutation and NPM1 mutation to predict survival and compared the result with that only using the score. Next, we used the multivariate logistic regression models that combined score and the above clinical indicators to assess the predictive performance. Finally, to test whether the MPG6 score could function as an independent prognostic factor, a multivariate survival analysis combined the above factors with multivariate Cox regression model was performed. The multivariate Cox regression analysis was performed in TCGA (n = 79) and GSE6891 (n = 187) CN-AML cohorts that had more detailed clinical information. Furthermore, we calculated the prediction error curve of the multivariate Cox regression model with R package “pec” 21. The prediction error curve was defined via Brier's score 22 and designed to estimate the performance of a risk prediction model.

### Benchmark the performance of MPG6

To evaluate the performance of MPG6, we benchmarked it with other three AML scoring models including the LSC17 score 23, the 7-gene score 24 and the 6-gene score 25 on CN-AML cohorts mentioned above. Survival analysis was performed using median score of each model as the threshold. For each model, absent genes in datasets were discarded.

## Results

### Identification of the independent prognostic MPG6 score in CN-AML

To identify potential independent prognostic markers in CN-AML, we initiated our study on the TCGA CN-AML cohort (n = 79). By performing Kaplan Meier analysis, we identified eight prognostic clinical indicators (age, sex, WBC count, mutation status and FAB classification) (p <0.1) (**Table S1**) and 1301 genes whose expression were significantly associated to OS. Then, after univariate and multivariate Cox regression analysis, we identified 203 genes independent of the eight clinical indicators in the TCGA CN-AML cohort (p <0.05) (**Table S2**). Among them, 23 were membrane protein genes, which are *AGPAT4*, *AMICA1*, *B4GALT7*, *BAIAP3*, *CCT6B*,* CD1C*, *CD1E*, *CD2*, *CD33*, *CD52*, *CD7*, *CD96*, *CRIM1*, *EMP1*, *GPR125*, *GPR153*, *HRH2*, *LTB*, *MBTPS1*, *SMAGP*, *STAB1*, *TREML2* and *TSPAN2*. After correlation analysis and linear regression analysis, we finally constructed the regression model: score = (0.0492 * *CD52*) - (0.0018 * CD96) + (0.0131 * *EMP1*) + (0.2058 * *TSPAN2*) + (0.0234 * *STAB1*) - (0.3658 * *MBTPS1*), which was named as MPG6 score (6-Membrane Protein Gene score). The multivariable analysis of six membrane protein genes and clinical variables in TCGA were list in Table 1. Meanwhile, those six membrane protein genes are well reported. Among them, *CD52*, a small glycoprotein that is linked by a glycosylphosphatidylinositol (GPI) anchor to the surface membrane and was reported as a prognostic marker in hematological malignancies 26. *CD96* is a membrane bound receptor of the immunoglobulin superfamily and belongs to a network of interactions that manipulates in a multifaceted fashion adhesion, activation, and inhibition of participating cells^27^. *EMP1* is an integral transmembrane glycoprotein, which has been identified as a poor prognostic factor in human cancers such as pediatric acute lymphoblastic leukemia, gliomas, gastric cancer, etc.^28^. *TSPAN2* is a cell surface membrane protein of the tetraspanin superfamily and is involved in tumor metastasis and invasiveness in human malignancy 29. *STAB1* encodes a multifunctional type I transmembrane protein, which was identified as a prognostic factor for CN-AML in our recent work 30. *MBTPS1* (also known as Golgi-resident site-1 protease, *S1P*) acts as the inactive type I membrane precursor protein and serve as a crucial component that catalyzing the initial, sterol-regulated cut in the luminal loops of sterol regulatory element (SRE)-binding proteins 31.

### High MPG6 score was correlated to poor survival in multiple independent datasets

**Figure 1:**
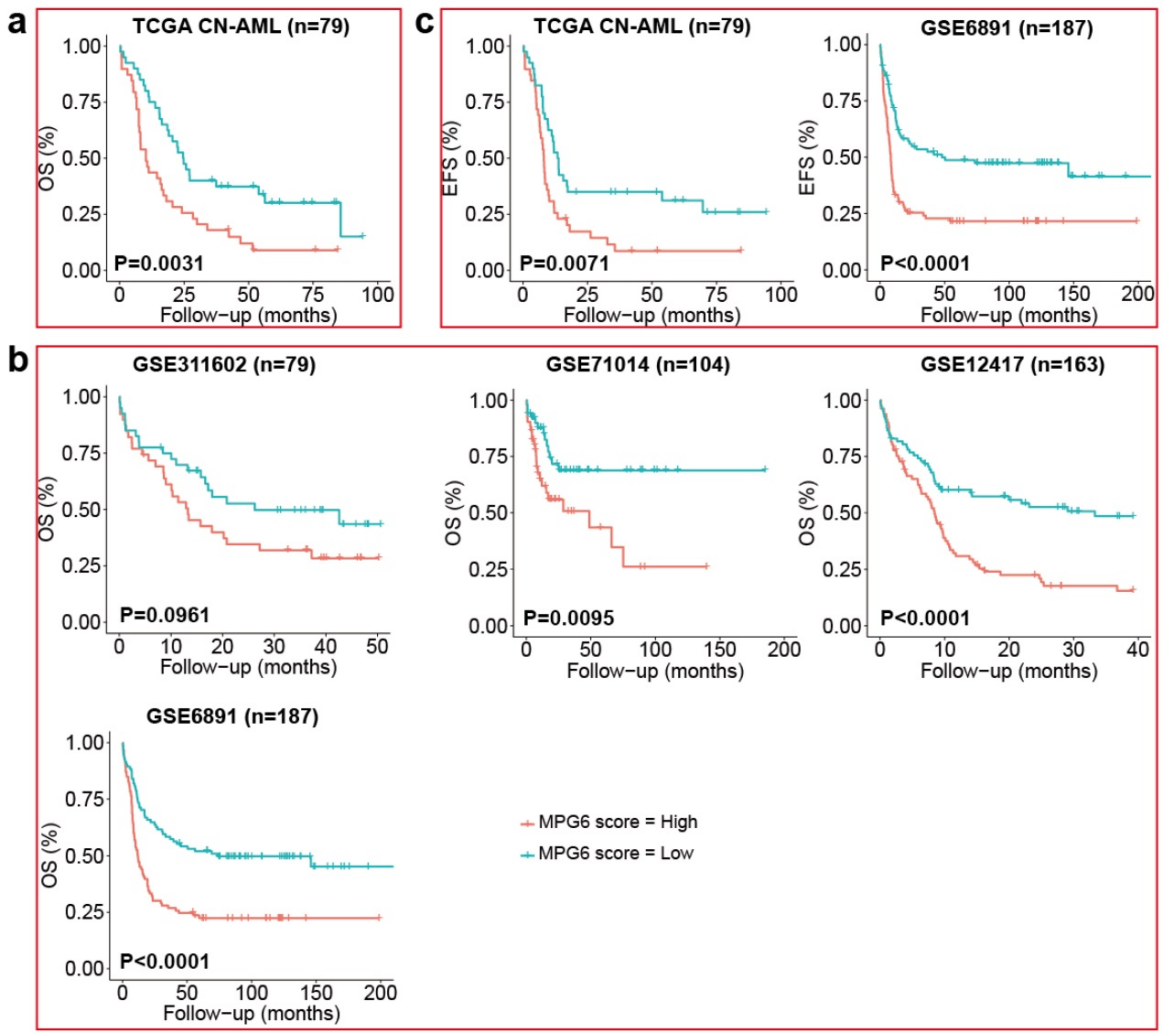
** The MPG6 score correlated to OS and EFS in five independent CN-AML cohort. (a)** Kaplan-Meier estimate of OS using median threshold of score in the TCGA CN-AML training dataset. **(b)** Kaplan-Meier estimate of OS using median threshold of score in other four array datasets (GSE311602, GSE71014, GSE6891 datasets). **(c)** Kaplan Meier-estimates of EFS using median threshold of score in TCGA CN-AML and GSE6891 datasets.

Survival analysis using the median of MPG6 score as threshold indicated that CN-AML patients with high MPG6 score generally had lower survival rate in the TCGA training cohort (**Fig. 1a**) as well as other four independent cohorts (**Fig. 1b**). Furthermore, the OS median value of high MPG6 score group was much lower than that of low score group in all five cohorts, as also did the numbers of patients with OS >2 years and OS >3 years (**Table 2**). Especially in the GSE71014 cohort (n = 104), patients in high MPG6 score group had significantly shorter OS than patients in low MPG6 score group (p = 0.0095) (**Fig. 1b**). In GSE6891 (n = 187) cohort, the median OS of low MPG6 score group was five times higher than that of high MPG6 score group (11.99 vs 65.25) (log rank test p <0.0001) (**Table 2**). The results indicated that the MPG6 score could serve as a prognostic factor in CN-AML.

Furthermore, we benchmarked the performance of MPG6 score with other scoring models including LSC17 score 23, the 7-gene score 24 and the 6-gene score 25 as we mentioned in the method. The result indicated that other scoring models were correlated to survival in only one or two datasets (**Fig. S1**), while the MPG6 score achieved robust performance with a significant correlation of survival in four of five datasets (**Fig. 1b**).

### The MPG6 score was significantly associated with patient clinical information

To further investigate the correlation of MPG6 score with other clinical indicators, we separated cohorts based on clinical information into high and low score groups, and the difference between two groups was measured by significance test for each clinical indicator. The result showed that except for OS, the MPG6 score was significantly associated with other clinical indicators such as WBC count (Wilcoxon rank-sum test p = 0.0053), EFS month (log rank test p = 0.0071 in TCGA CN-AML cohort and p <0.0001 in GSE6891 cohort) (**Fig. 1c**) and the presence of FLT3-ITD mutation (Fisher's exact test p = 0.0248) (**Table 3**). In addition, patients in high MPG6 score group was observed with higher WBC (median 50.64 vs 5) and shorter EFS than in low MPG6 score group (median 7.2 vs 13.4 in TCGA CN-AML cohort and 9 vs 14.39 in GSE6891 cohort) (log rank test p <0.0001) (**Table 3**). In GSE6891 cohort, the ratio of FLT3-ITD positive patients in high score group was 52.13% (49 out of 94) and 31.18% (29 out of 93) in low MPG6 score groups (Fisher's exact test p = 0.0248) (**Table 3**).

### The MPG6 score can improve the predictive performance to predict survival of patients

**Figure 2:**
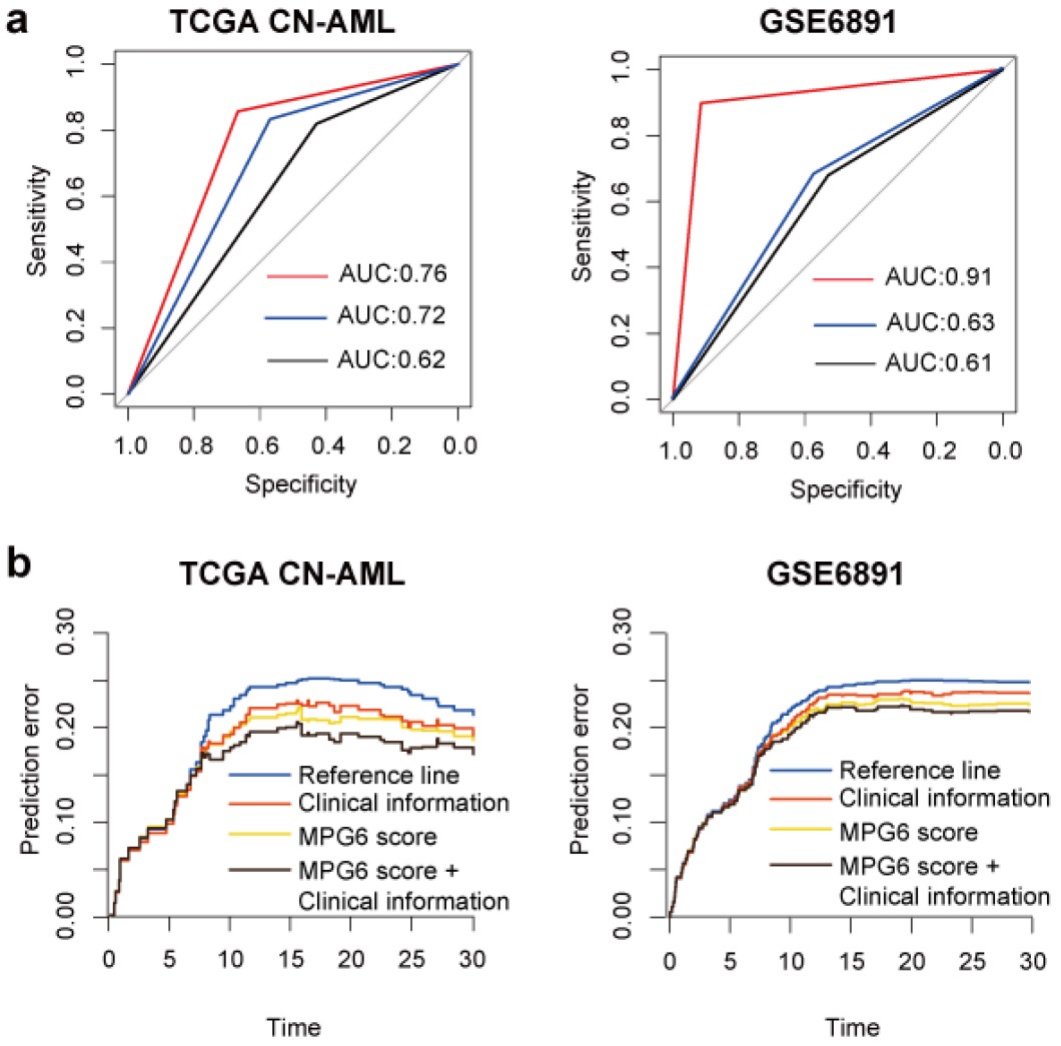
** The MPG6 score can improve the performance to predict survival. (a)** ROC curve of logistic regression model in TCGA CN-AML and GSE6891 cohorts. The red line represents the ROC curve of combining MPG6 score with clinical information including age, WBC count, FLT3-ITD mutation and NPM1 mutation as features; the blue line represents ROC curve of using MPG6 score as the only feature in logistic regression model, and the black line represents ROC curve using clinical information mentioned above as features. **(b)** Prediction error curve of multivariate Cox regression model with or without MPG6 score. Reference line indicates Kaplan-Meier estimation without additional variables. Lower curve (lower prediction error) indicates better predictive value.

To investigate the performance of MPG6 score in predicting survival of patients, we performed logistic regression using MPG6 score as a single variant in the model and compared the result with models (detail in method). The result showed that in the training cohort, the predictive performance of MPG6 score as a single continuous variant outperformed the performance of combined variants including age, WBC count, FLT3-ITD and NPM1 mutation (AUC = 0.702 versus 0.624) (**Fig. 2a**). In multivariate logistic regression models that considered age, WBC count, FLT3-ITD mutation, NPM1 mutation and score as variants, we observed that the inclusion of MPG6 score greatly improved the predictive performance (AUC = 0.762 versus 0.624 in TCGA CN-AML cohort and AUC = 0.912 versus 0.614 in GSE6891 cohort) (**Fig. 2a**). The results demonstrated that the MPG6 score improved the performance to predict survival of CN-AML patients.

### The MPG6 score can function as a good independent prognostic factor

To investigate whether the performance of MPG6 score affected by other known predictors of outcome, we performed multivariate survival analysis based on the multivariate Cox regression model. The result indicated that the score could serve as an independent prognostic factor in two tested cohorts with detailed clinical information available including TCGA and GSE6891 (**Table 4**). Besides, we observed that after the inclusion of score in the model, some known predictors of outcome turned to be not significant, such as the WBC count in TCGA CN-AML cohort. However, the presence of NPM1 mutation was not significant no matter with or without the score in the model (**Table 4**). The result demonstrated that MPG6 score can be an independent prognostic factor and outperformed other known predictors. In addition, the prediction error curve indicated that MPG6 score could improve the predictive performance of multivariate Cox regression model (**Fig. 2b**).

## Discussion

Over the past decades, the high heterogeneity of CN-AML presents a considerable challenge in the risk stratification 32. About 20-30% of genes encode membrane proteins, which have immense significance in pharmacological research. However, very few studies about their potential as prognostic indicators in leukemia were conducted. Therefore, our study aimed at searching membrane protein genes to stratify CN-AML patients and predict the outcome of CN-AML. In this study, we generated a 6-membrane protein gene (MPG6) score from the TCGA dataset and confirmed in four independent validation sets (**Fig. 1**). Among them, many were confirmed as the therapeutic targets in leukemia. CD52 has been developed as a drug target in chronic lymphocytic leukemia (CLL) 33 and predicted to be a prognostic marker in AML 34. CD96 may serve as an LSC-specific therapeutic target 35. *EMP1* was identified as a potential drug target in acute lymphoblastic leukemia (ALL) 36. Although the role of *TSPAN2*,* STAB1* and *MBTPS1*were not confirmed in leukemia, they were reported to be involved in the progression of the tumor metastasis^37^-^39^. From other point of view, our strategy to identify MPG6 demonstrated a credible approach, which may also be applicable in identifying such gene signatures in other types of cancers.

To our knowledge, this is the first report about predictive and/or prognostic biomarkers related to membrane protein gene in CN-AML. Although some prognostic biomarkers or factors of CN-AML patients were proven to be valuable, only a part of them was applied in clinical trial. Moreover, the prognostic values of many markers appear to be controversial because they were validated in limited samples. Compared with above issues, the MPG6 made up of only six genes and were easy testing using qPCR. Nevertheless, the limitation of MPG6 was that we detected mRNA expression here not protein. Therefore, a further detection of the membrane protein expression such as flow cytometry may be necessary.

It was reported that the outcome of AML was correlated to FLT3-ITD mutation and NPM1 mutation. The patients with FLT3-ITD mutation positive have a generally poor prognosis 40, on the contrary, patients with NPM1 mutation positive generally have a good outcome 41. In this work, we expect that there are some correlations between gene mutations and MPG6. However, we only found that FLT3-ITD mutations were significantly associated with the high MPG6 score group in GSE6891 dataset (**Table 3**). Meanwhile, we observed that compared with OS, the correlation of MPG6 with other clinical indicators were not that strong, such as the difference of WBC count and FLT3-ITD mutation between high- and low-score groups were significant only in certain tested dataset. We consider that this may be caused by the following reason, the CN-AML was highly heterozygous, though, the model trained with genes' expression against survival performs good in predicting survival, when it came to the detail clinical indicators such as WBC count and FLT3-TID mutation, it worked not that well. In view of this problem, we suggest in the future work related to the prognostic model in CN-AML, clinical indicators related to the prognostic could be considered into the training model, which may lead to a more robust result.

## Supplementary Material

Supplementary figures and tables.

## Figures and Tables

**Table 1:** Multivariable analysis of six membrane protein genes in TCGA cohort.

	HR (95% CI)/(p-value)					
OS Covariate	*CD52*	*CD96*	*EMP1*	*TSPAN2*	*STAB1*	*MBTPS1*
Age	2.42	2.59	2.36	2.25	2.44	1.59
	(1.13-3.67) /(p=0.013)	(1.27-3.99)/(p=0.0011)	(1.07-3.51)/(p=0.0104)	(1.03-3.32)/(p=0.0172)	(1.15-3.62)/(p=0.0147)	(1.04-3.18)/(p=0.0077)
DNMT3A	1.45	2.63	2.21	2.53	2.20	2.07
	(0.69-2.45)/(p=0.1053)	(1.27-3.96)/(p=0.1008)	(1.07-3.43)/(p=0.018)	(1.08-4.16)/(p=0.0069)	(1.07-3.50)/(p=0.028)	(1.02-3.27)/(p=0.019)
RUNX1	1.91	2.14	1.97	1.82	2.04	1.91
	(0.92-3.06)/(p=0.0329)	(1.09-5.82)/(p=0.0488)	(0.78-4.39)/(p=0.1763)	(0.79-4.83)/(p=0.0574)	(1.03-5.37)/(p=0.0417)	(0.88-4.57)/(p=0.0407)
FLT3-ITD	-1.47	0.64	1.74	1.53	1.59	1.57
	(0.12-1.23)/(p=0.1377)	(0.67-2.48)/(p=0.5326)	(0.86-3.13)/(p=0.0517)	(0.79-3.01)/(p=0.1103)	(0.89-3.07)/(p=0.1124)	(0.77-2.97)/(p=0.1074)
MT-CYB	1.76	1.71	1.25	1.04	1.33	1.32
	(0.93-4.57)/(p=0.0729)	(0.82-10.94)/(p=0.1338)	(0.63-8.51)/(p=0.3494)	(0.53-7.42)/(p=0.2805)	(0.66-8.75)/(p=0.185)	(0.58-7.75)/(p=0.175)
WT1	1.54	0.77	0.72	0.57	0.16	0.17
	(0.67-10.11)/(p=0.1203)	(0.58-3.92)/(p=0.4993)	(0.53-3.95)/(p=0.4718)	(0.48-3.60)/(p=0.5357)	(0.44-5.02)/(p=0.2461)	(0.43-4.92)/(p=0.2031)
IDH2	0.07	0.021	0.43	0.52	-0.47	-0.46
	(0.43-2.26)/(p=0.9125)	(0.51-2.48)/(p=0.098)	(0.53-2.65)/(p=0.4344)	(0.56-2.57)/(p=0.5327)	(0.56-1.85)/(p=0.638)	(0.55-1.75)/(p=0.535)
NPM1	0.68	0.19	0.16	0.19	-0.05	-0.04
	(0.52-3.79)/(p=0.4357)	(0.59-2.01)/(p=0.8976)	(0.51-1.86)/(p=0.817)	(0.57-2.07)/(p=0.7654)	(0.53-1.85)/(p=0.963)	(0.51-1.82)/(p=0.768)
IDH1	-0.23	-1.47	-1.31	-1.43	-1.28	-1.18
	(0.41-1.67)/(p=0.7176)	(0.13-1.23)/(p=0.1287)	(0.2-1.28)/(p=0.144)	(0.12-1.27)/(p=0.145)	(0.16-1.4)/(p=0.2016)	(0.14-1.2)/(p=0.1789)
Total	2.4	2.5	2.2 (	1.8	1.98	0.56
	(1.2-4.8)/(p=0.013)	(1.2-5.2)/(p=0.016)	1.2-4.1)/(p=0.018)	(1-3.3)/(p=0.041)	(1.01-3.03)/(p=0.0473)	(0.32-0.98)/(p=0.041)

The model was generated from a Cox regression model that included Age, gene mutation of DNMT3A, and RUNX1, FLT3-ITD, MT-CYB, WT1, IDH2, NPM1, IDH1 and expression level of each membrane protein gene. HR: Hazard Ratio.

**Table 2:** Overall survival (OS) in high score and low score groups of five datasets.

Dataset	Race	OS	HS group	LS group
TCGA CN-AML (n=79)	White/Africa	OS median (months)	10.45	24.8
		Number of OS>2 years	11	21
		Number of OS>3 years	7	15
GSE311602 (n=79)	White	OS median	12.17	17.97
		Number of OS>2 years	13	18
		Number of OS>3 years	11	13
GSE71014 (n=104)	White	OS median	9	21.9
		Number of OS>2 years	12	25
		Number of OS>3 years	8	17
GSE12417 (n=163)	Asian	OS median	8.18	14.03
		Number of OS>2 years	15	31
		Number of OS>3 years	9	22
GSE6891 (n=187)	White	OS median	11.99	65.25
		Number of OS>2 years	29	60
		Number of OS>3 years	26	54

HS: High Score; LS: Low Score.

**Table 3:** Clinical characteristics of the TCGA CN-AML and GSE6891 cohorts.

Clinical information	TCGA CN-AML cohort			GSE6891 cohort		
	High score	Low score	p-value	High score	Low score	p-value
OS median	10.45	24.8	0.0031$	11.99	65.25	0.000015$
Sex (number)	M: 18	M: 21	0.8600+	M: 15	M: 20	0.0825+
	F: 22	F:18		F: 24	F: 19	
Age (median)	66	68	0.2675*	48	45	0.7201*
BM blast (%)	77.5	66	0.3798||	-	-	-
WBC count	50.54	5	0.0053||	-	-	-
NPM1 mutation	Pos: 23	Pos: 17	0.2635+	Pos: 57	Pos: 48	0.2399+
	Neg: 17	Neg: 22		Neg: 37	Neg: 45	
FLT3 mutation	Pos: 17	Pos: 12	0.3524+	Pos: 49	Pos: 29	0.0248+
	Neg: 23	Neg: 27		Neg: 45	Neg: 64	
EFS month	7.2	13.4	0.0071$	9.00	14.39	<0.0001$

Pos: Positive; Neg: Negative; $: log rank test; *: Student's t-test; +: Fisher's exact test; ||: Wilcoxon rank-sum test; The “-” in table means clinical indicators were absent in GSE6891 cohort.

**Table 4:** Multivariate survival analysis of score and other known predictors of outcome in TCGA CN-AML and GSE6891 cohorts.

Overall Survival Covariate	TCGA CN-AML cohort		GSE6891 cohort	
	Hazard Ratio (95% CI)	p-value	Hazard Ratio (95% CI)	p-value
Age (median)	1.02 (1.00-1.04)	0.0474	1.00 (0.99-1.02)	0.6986
WBC count	1.00 (0.99-1.01)	0.1361	-	-
BM blast (%)	0.99 (0.97-1.01)	0.1707	-	-
NPM1 mutation	0.88 (0.46-1.66)	0.6835	0.66 (0.45-0.97)	0.0366
FLT3 mutation	2.03 (1.11-3.73)	0.0216	1.55 (1.04-2.30)	0.0296
MPG6 score	1.08 (1.03-1.12)	0.0004	4.12 (2.04-8.30)	<0.0001

The “-” in table means clinical indicators were absent in GSE6891 cohort.

## Abbreviations

ALL: acute lymphoblastic leukemia
AML: acute myeloid leukemia
CI: confidence interval
CLL: chronic lymphocytic leukemia
CN-AML: cytogenetically normal acute myeloid leukemia
CPH: Cox proportional hazards
OS: overall survival
MPG6: 6-Membrane Protein Gene
TCGA: The Cancer Genome Atlas
WBC: white blood cell
HR: Hazard ratios
