# Supplementary material for: A 6-Membrane Protein Gene score for prognostic prediction of cytogenetically normal acute myeloid leukemia in multiple cohorts
Source: J Cancer. 2020 Jan 1;11(1):251–9. doi: 10.7150/jca.35382 (PMC6930412; doi:10.7150/jca.35382)
Supplement: Supplementary file 1 — Supplementary figures and tables. [file jcav11p0251s1.pdf]

Supporting Information

Table S1. Prognostic clinical indicators in univariate survival analysis in TCGA dataset.

Clinical Index	beta	HR (95% CI for HR)	wald.test	p.value
Sex.	-0.31	0.74 (0.45-1.2)	1.5	0.23
Age.	0.75	2.1 (1.3-3.5)	8.7	**0.0032**
WBC.	0.24	1.3 (0.74-2.2)	0.76	0.38
BM_Blast.	-0.0056	0.99 (0.98-1)	0.66	0.42
PML_RARA.	0	1 (1-1)	NA	NA
MLL_partner.	0	1 (1-1)	NA	NA
MYH11_CBFB.	0	1 (1-1)	NA	NA
RUNX1_RUNX1T1.	0	1 (1-1)	NA	NA
MLLT10_partner.	0	1 (1-1)	NA	NA
BCR_ABL.	0	1 (1-1)	NA	NA
GPR128_TFG.	0	1 (1-1)	NA	NA
NUP98_NSD1.	-1.8	0.17 (0.038-0.74)	5.6	**0.018**
MLL_PTD.	0.48	1.6 (0.76-3.4)	1.6	0.21
FLT3.	-0.46	0.63 (0.38-1.1)	3.1	**0.078**
NPM1.	0.15	1.2 (0.71-1.9)	0.37	0.54
DNMT3A.	-0.44	0.64 (0.39-1.1)	2.8	**0.093**
IDH2.	-0.39	0.68 (0.34-1.3)	1.2	0.27
IDH1.	1.2	3.2 (1.2-8.9)	5	**0.026**
RUNX1.	-0.58	0.56 (0.28-1.1)	2.8	**0.097**
TET2.	0.45	1.6 (0.75-3.3)	1.4	0.23
TP53.	-0.95	0.39 (0.052-2.9)	0.87	0.35
NRAS.	0.9	2.4 (0.77-7.8)	2.3	0.13
CEBPA.	0.14	1.2 (0.5-2.7)	0.11	0.74
WT1.	-0.1	0.9 (0.36-2.3)	0.05	0.83
PTPN11.	-0.21	0.81 (0.33-2)	0.2	0.66
KIT.	0	1 (1-1)	NA	NA
KRAS.	0.032	1 (0.25-4.2)	0	0.96
MT_CO2.	-1.1	0.34 (0.081-1.4)	2.2	0.14
TTN.	-0.78	0.46 (0.19-1.1)	3.2	0.073
U2AF1.	17	2.6e+07 (0-Inf)	0	1
SMC1A.	-0.7	0.5 (0.18-1.4)	1.8	0.18
SMC3.	-0.74	0.48 (0.17-1.3)	2	0.16
STAG2.	0.16	1.2 (0.42-3.2)	0.09	0.76
MT_CYB.	-1.4	0.24 (0.072-0.81)	5.3	**0.022**
PHF6.	-0.18	0.84 (0.26-2.7)	0.09	0.77
FAB	NA	NA	5.02	0.54
FAB.M1	-0.84353	0.43 (0.14-1.3)	NA	0.140695
FAB.M2	-0.61088	0.54 (0.18-1.7)	NA	0.284304
FAB.M3	NA	NA	NA	NA
FAB.M4	-0.25097	0.78 (0.26-2.3)	NA	0.654013

FAB.M5	-0.80282	0.45 (0.13-1.6)	NA	0.211716
FAB.M6	NA	NA	NA	NA
FAB.M7	0.408307	1.5 (0.17-14)	NA	0.716368

Table S2. 203 genes independent of the eight clinical indicators in the TCGA CN-AML cohort (P<0.05).

Gene	beta	HR (95% CI for HR)	p.value
FAM64A	-1.1	0.32 (0.14-0.77)	0.01
KBTBD11	-1.1	0.34 (0.18-0.67)	0.0015
FBXO25	-1	0.35 (0.18-0.68)	0.002
ASCL2	-1	0.36 (0.19-0.66)	0.001
PCK2	-1	0.36 (0.2-0.64)	0.00054
CDCA5	-0.99	0.37 (0.2-0.67)	0.0011
AKAP2	-0.96	0.38 (0.17-0.88)	0.023
PALM2	-0.96	0.38 (0.17-0.88)	0.023
CRIM1	-0.93	0.4 (0.2-0.79)	0.0081
CPD	-0.88	0.41 (0.21-0.82)	0.011
FOXN3	-0.89	0.41 (0.21-0.82)	0.011
BEND3	-0.9	0.41 (0.22-0.76)	0.0045
LOC643837	-0.89	0.41 (0.22-0.76)	0.0045
APOD	-0.86	0.42 (0.22-0.83)	0.012
HES6	-0.87	0.42 (0.23-0.78)	0.0061
CDT1	-0.85	0.43 (0.23-0.79)	0.0071
CBX2	-0.84	0.43 (0.23-0.8)	0.0076
NNMT	-0.85	0.43 (0.24-0.78)	0.0051
ERAP2	-0.82	0.44 (0.25-0.77)	0.0042
LUM	-0.81	0.44 (0.25-0.79)	0.006
FLI1	-0.81	0.45 (0.24-0.82)	0.0094
CYL7	-0.8	0.45 (0.24-0.86)	0.015
CATSPER2P1	-0.81	0.45 (0.25-0.8)	0.0073
MTMR6	-0.79	0.45 (0.25-0.81)	0.008
CERK	-0.78	0.46 (0.22-0.96)	0.039
APOE	-0.78	0.46 (0.24-0.87)	0.017
ZNF491	-0.77	0.46 (0.26-0.81)	0.0068
FGD5	-0.75	0.47 (0.24-0.94)	0.034
TK1	-0.76	0.47 (0.26-0.83)	0.0093
DDIT4L	-0.75	0.47 (0.26-0.88)	0.018
P2RY8	-0.75	0.47 (0.27-0.84)	0.01
M6PR	-0.75	0.47 (0.28-0.81)	0.0066
CCL14	-0.74	0.48 (0.24-0.93)	0.03
IGFBP3	-0.74	0.48 (0.25-0.91)	0.024
LBR	-0.74	0.48 (0.25-0.92)	0.027
TROAP	-0.73	0.48 (0.26-0.88)	0.019

HIST1H3B	-0.74	0.48 (0.27-0.86)	0.014
CXCL12	-0.71	0.49 (0.26-0.92)	0.026
BGN	-0.72	0.49 (0.26-0.92)	0.027
MCM7	-0.72	0.49 (0.26-0.92)	0.027
DCN	-0.71	0.49 (0.27-0.92)	0.025
COL1A2	-0.69	0.5 (0.27-0.93)	0.028
CEBPA	-0.68	0.51 (0.27-0.95)	0.034
FSTL1	-0.67	0.51 (0.27-0.97)	0.041
DPY19L2	-0.68	0.51 (0.28-0.9)	0.021
MGP	-0.67	0.51 (0.28-0.96)	0.036
SLC24A3	-0.65	0.52 (0.28-0.96)	0.035
COL3A1	-0.65	0.52 (0.28-0.97)	0.04
PATZ1	-0.66	0.52 (0.29-0.93)	0.028
B4GALT7	-0.65	0.52 (0.29-0.96)	0.037
GFM2	-0.63	0.53 (0.29-0.97)	0.039
E2F1	-0.64	0.53 (0.29-0.97)	0.04
GPR153	-0.64	0.53 (0.29-0.97)	0.041
GPR125	-0.63	0.53 (0.29-0.99)	0.048
TGIF1	-0.63	0.53 (0.3-0.94)	0.028
RHOBTB3	-0.62	0.54 (0.29-0.98)	0.044
BIRC5	-0.62	0.54 (0.3-0.96)	0.037
EPR1	-0.62	0.54 (0.3-0.96)	0.037
FAM178B	-0.61	0.54 (0.3-0.97)	0.038
ZC3H4	-0.61	0.54 (0.3-0.97)	0.038
RNF26	-0.61	0.54 (0.3-0.97)	0.041
FOXM1	-0.61	0.54 (0.3-0.99)	0.046
FOXK1	-0.62	0.54 (0.31-0.92)	0.025
GTSE1	-0.61	0.54 (0.31-0.96)	0.036
ORC1	-0.6	0.55 (0.31-0.97)	0.038
FLT4	-0.6	0.55 (0.31-0.97)	0.04
HIST2H2AC	-0.59	0.55 (0.31-1)	0.05
BRD3	-0.59	0.56 (0.32-0.97)	0.04
MBTPS1	-0.58	0.56 (0.32-0.98)	0.041
CP110	-0.57	0.56 (0.32-0.99)	0.047
SPC24	-0.58	0.56 (0.32-0.99)	0.047
NCAPH	-0.57	0.56 (0.33-0.97)	0.04
EIF4EBP2	-0.57	0.57 (0.33-0.98)	0.04
RFESD	-0.56	0.57 (0.33-0.99)	0.048
RETN	0.55	1.7 (1-3)	0.045
SNORA76	0.56	1.7 (1-3)	0.046
ACSF2	0.59	1.8 (1.1-3.1)	0.032
PRKRIP1	0.59	1.8 (1.1-3.1)	0.032
ZRSR2	0.61	1.8 (1.1-3.2)	0.027
MRPS18C	0.61	1.8 (1.1-3.2)	0.028

NUDT8	0.58	1.8 (1-3)	0.033
ARHGAP27	0.59	1.8 (1-3.1)	0.035
AMPH	0.58	1.8 (1-3.1)	0.038
FLJ45244	0.59	1.8 (1-3.1)	0.04
HTR7	0.57	1.8 (1-3.1)	0.046
PDK2	0.57	1.8 (1-3.1)	0.046
MLANA	0.57	1.8 (1-3.1)	0.05
HRH2	0.6	1.8 (1-3.2)	0.033
STAB1	0.6	1.8 (1-3.2)	0.038
TXNDC6	0.6	1.8 (1-3.2)	0.038
KIAA0125	0.59	1.8 (1-3.2)	0.045
NUDT17	0.61	1.8 (1-3.3)	0.039
TSPAN2	0.61	1.8 (1-3.3)	0.041
PLEKHB1	0.61	1.8 (1-3.4)	0.05
CSF1R	0.64	1.9 (1.1-3.2)	0.015
TFPT	0.64	1.9 (1.1-3.2)	0.019
C2orf24	0.63	1.9 (1.1-3.2)	0.021
DUSP3	0.63	1.9 (1.1-3.2)	0.022
AMICA1	0.62	1.9 (1.1-3.2)	0.025
ISCU	0.62	1.9 (1.1-3.2)	0.027
VIM	0.66	1.9 (1.1-3.3)	0.017
BAIAP3	0.64	1.9 (1.1-3.3)	0.026
LARP7	0.64	1.9 (1.1-3.3)	0.026
ABLIM1	0.63	1.9 (1.1-3.3)	0.032
XPR1	0.63	1.9 (1.1-3.3)	0.032
SLC7A6OS	0.64	1.9 (1.1-3.4)	0.033
IL32	0.66	1.9 (1.1-3.5)	0.031
DNAJB2	0.62	1.9 (1-3.3)	0.034
GABBR1	0.63	1.9 (1-3.4)	0.042
SOCS2	0.63	1.9 (1-3.4)	0.043
C17orf108	0.62	1.9 (1-3.4)	0.047
IL2RA	0.64	1.9 (1-3.5)	0.039
NCALD	0.63	1.9 (1-3.5)	0.04
CCT6B	0.64	1.9 (1-3.5)	0.044
PTH2R	0.65	1.9 (1-3.6)	0.04
TPPP3	0.67	2 (1.1-3.4)	0.017
TNFRSF25	0.67	2 (1.1-3.4)	0.018
PXN	0.69	2 (1.1-3.5)	0.015
ABCC2	0.7	2 (1.1-3.5)	0.016
TRIM71	0.69	2 (1.1-3.5)	0.016
CMKLR1	0.69	2 (1.1-3.5)	0.02
KMO	0.68	2 (1.1-3.5)	0.02
CCDC137	0.68	2 (1.1-3.5)	0.023
FAM118B	0.7	2 (1.1-3.6)	0.015

PDLIM7	0.71	2 (1.1-3.6)	0.017
GEMIN7	0.69	2 (1.1-3.6)	0.025
ATP13A2	0.71	2 (1.1-3.7)	0.018
STARD4	0.71	2 (1.1-3.7)	0.021
FAM63B	0.7	2 (1.1-3.7)	0.025
LTB	0.7	2 (1.1-3.7)	0.026
CD33	0.7	2 (1.1-3.8)	0.027
TPT1	0.71	2 (1.2-3.5)	0.011
PDLIM1	0.68	2 (1-3.7)	0.037
RDH13	0.67	2 (1-3.7)	0.043
CD1C	0.69	2 (1-3.8)	0.036
PIK3R6	0.67	2 (1-3.8)	0.045
SAMD3	0.67	2 (1-3.8)	0.047
CD1E	0.67	2 (1-3.8)	0.049
EOMES	0.7	2 (1-3.9)	0.04
SPAG1	0.7	2 (1-3.9)	0.043
EPSTI1	0.72	2 (1-4)	0.036
C1orf124	0.73	2.1 (1.1-3.8)	0.017
DAAM1	0.74	2.1 (1.1-3.9)	0.02
ELN	0.74	2.1 (1.1-3.9)	0.02
LRRK1	0.75	2.1 (1.1-4)	0.02
CD2	0.74	2.1 (1.1-4)	0.023
ECE1	0.73	2.1 (1.1-4.1)	0.035
ME1	0.72	2.1 (1.2-3.5)	0.0086
DGKA	0.73	2.1 (1.2-3.6)	0.0098
CEP164	0.75	2.1 (1.2-3.7)	0.0085
PRSS2	0.73	2.1 (1.2-3.7)	0.014
ZNF133	0.73	2.1 (1.2-3.7)	0.015
C1orf56	0.75	2.1 (1.2-3.8)	0.0094
PLCD3	0.76	2.1 (1.2-3.8)	0.0094
ADK	0.75	2.1 (1.2-3.8)	0.013
CCDC75	0.74	2.1 (1.2-3.8)	0.013
IL17RC	0.76	2.1 (1-4.4)	0.04
RASGEF1A	0.77	2.2 (1.1-4)	0.018
BAI1	0.77	2.2 (1.1-4.3)	0.028
FOXO1	0.78	2.2 (1.1-4.5)	0.035
LRG1	0.8	2.2 (1.1-4.7)	0.034
PRMT2	0.77	2.2 (1.2-3.8)	0.0071
IL1RAP	0.77	2.2 (1.2-3.8)	0.0079
TNFRSF18	0.78	2.2 (1.2-3.9)	0.0091
CD7	0.8	2.2 (1.2-4)	0.0077
SOCS1	0.79	2.2 (1.2-4)	0.0087
PHLDB3	0.79	2.2 (1.2-4)	0.0095
EMP1	0.79	2.2 (1.2-4.1)	0.013

AADAT	0.81	2.2 (1.3-4)	0.0056
TLR9	0.8	2.2 (1.3-4)	0.0066
HSD17B7	0.84	2.3 (1.1-4.8)	0.025
ROBO4	0.82	2.3 (1.2-4.3)	0.013
RNF144B	0.81	2.3 (1.3-4)	0.0045
CPA6	0.85	2.3 (1.4-4.1)	0.0024
PLEKHA6	0.87	2.4 (1.2-4.6)	0.0086
CD52	0.88	2.4 (1.2-4.8)	0.013
CLIC3	0.87	2.4 (1.3-4.2)	0.003
HAT1	0.87	2.4 (1.3-4.2)	0.0031
LOC729156	0.87	2.4 (1.3-4.2)	0.0032
SNORD42A	0.87	2.4 (1.3-4.2)	0.0035
LCN6	0.89	2.4 (1.3-4.4)	0.0036
NCRNA00241	0.87	2.4 (1.3-4.4)	0.0058
HEXIM1	0.86	2.4 (1.3-4.4)	0.0066
GOLGA8B	0.88	2.4 (1.3-4.5)	0.0052
AGPAT4	0.89	2.4 (1.3-4.6)	0.0061
ETS2	0.87	2.4 (1.4-4.2)	0.0021
USE1	0.88	2.4 (1.4-4.2)	0.0022
PRDM16	0.92	2.5 (1.2-5.1)	0.012
CD96	0.91	2.5 (1.2-5.2)	0.016
AHNAK2	0.93	2.5 (1.2-5.5)	0.017
SNORA48	0.92	2.5 (1.4-4.4)	0.0011
XPC	0.92	2.5 (1.4-4.5)	0.0019
MREG	0.9	2.5 (1-5.9)	0.042
ADAMTS10	0.94	2.6 (1.3-5.2)	0.0085
WFDC1	0.94	2.6 (1.4-4.8)	0.0034
IL3RA	0.95	2.6 (1.5-4.5)	0.001
SMAGP	0.98	2.7 (1.4-5.1)	0.0028
FGFBP2	0.98	2.7 (1.5-4.9)	0.0015
PTPRM	1.1	2.9 (1.5-5.6)	0.0021
SUSD3	1.1	3.1 (1.5-6.3)	0.0019
UTP6	1.2	3.2 (1.4-7.1)	0.0046
CYP2C8	1.2	3.4 (1.8-6.5)	0.00026
TREML2	1.3	3.6 (1.9-6.9)	0.00012

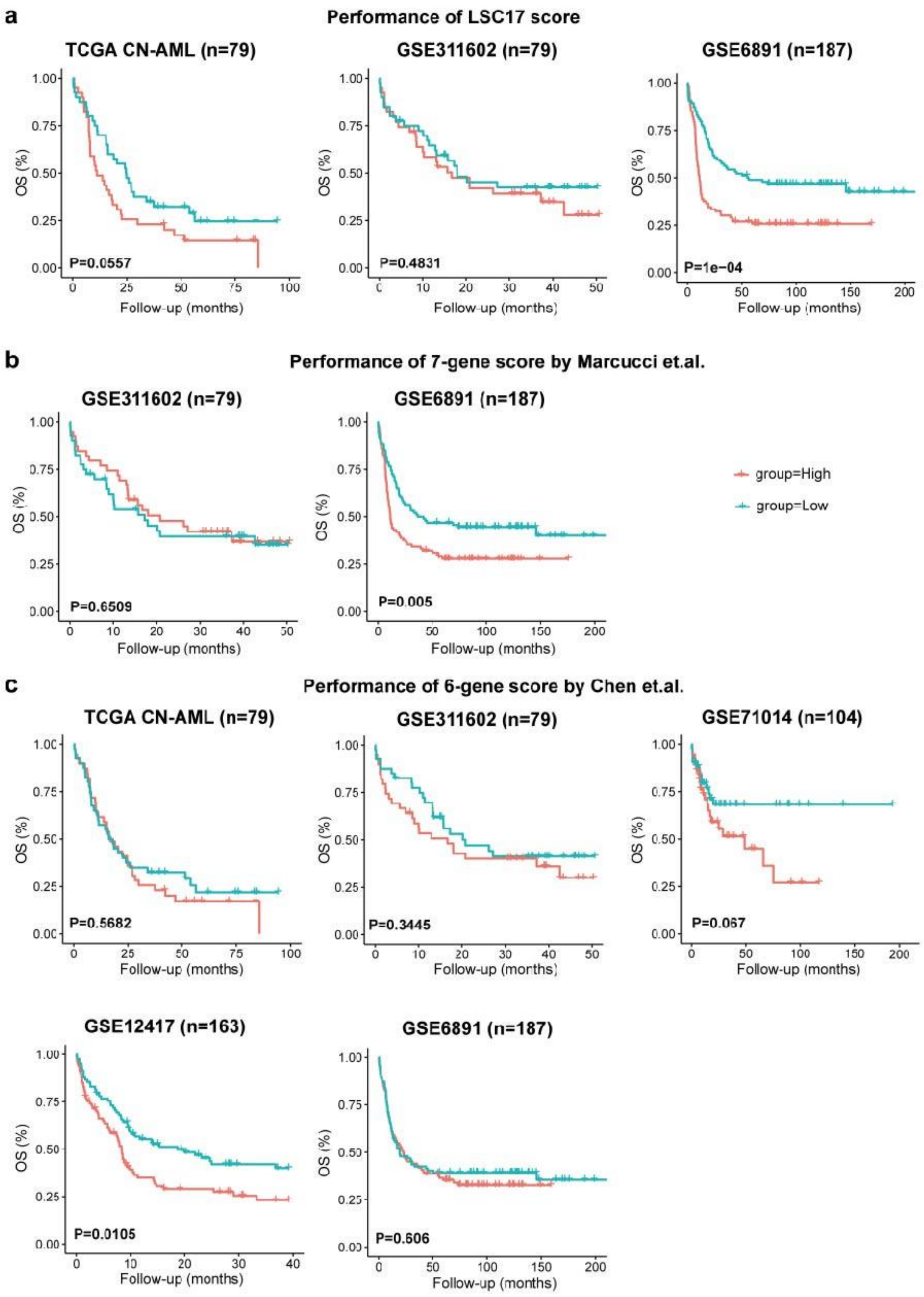

Fig. S1 Performance of other scoring models in CN-AML cohorts

a. Kaplan–Meier estimates of OS using median threshold of LSC17 score in three datasets with expression of genes in LSC17 score available.

b. Kaplan–Meier estimates of OS using median threshold of 7-gene score by Marcucci et.al. in two datasets with expression of genes in the scoring model available.

c. Kaplan–Meier estimates of OS using median threshold of 6-gene score by Chen et.al. in five datasets with expression of genes in the scoring model available.
